# Cytotoxic effects and tolerability of gemcitabine and axitinib in a xenograft model for c-myc amplified medulloblastoma

**DOI:** 10.1038/s41598-021-93586-x

**Published:** 2021-07-07

**Authors:** Stefanie Schwinn, Zeinab Mokhtari, Sina Thusek, Theresa Schneider, Anna-Leena Sirén, Nicola Tiemeyer, Ignazio Caruana, Evelina Miele, Paul G. Schlegel, Andreas Beilhack, Matthias Wölfl

**Affiliations:** 1 Children’s Hospital, Pediatric Hematology, Oncology and Stem Cell Transplantation, Würzburg University Hospital, 31, Josef-Schneider-Str. 2, 97080 Würzburg, Germany; 2 Department of Medicine, II, Würzburg University Hospital, Zinklesweg 10, 97078 Würzburg, Germany; 3 Department of Neurosurgery, Würzburg University Hospital, Würzburg, Germany; 4 Comprehensive Cancer Center Main-Franken, Würzburg University Hospital, Würzburg, Germany; 5 Department of Pediatric Onco-Hematology, Cell and Gene Therapy, Bambino Gesù Children’s Hospital, IRCCS, Rome, Italy

**Keywords:** Paediatric cancer, Cancer, CNS cancer

## Abstract

Medulloblastoma is the most common high-grade brain tumor in childhood. Medulloblastomas with c-myc amplification, classified as group 3, are the most aggressive among the four disease subtypes resulting in a 5-year overall survival of just above 50%. Despite current intensive therapy regimens, patients suffering from group 3 medulloblastoma urgently require new therapeutic options. Using a recently established c-myc amplified human medulloblastoma cell line, we performed an in-vitro-drug screen with single and combinatorial drugs that are either already clinically approved or agents in the advanced stage of clinical development. Candidate drugs were identified in vitro and then evaluated in vivo. Tumor growth was closely monitored by BLI. Vessel development was assessed by 3D light-sheet-fluorescence-microscopy. We identified the combination of gemcitabine and axitinib to be highly cytotoxic, requiring only low picomolar concentrations when used in combination. In the orthotopic model, gemcitabine and axitinib showed efficacy in terms of tumor control and survival. In both models, gemcitabine and axitinib were better tolerated than the standard regimen comprising of cisplatin and etoposide phosphate. 3D light-sheet-fluorescence-microscopy of intact tumors revealed thinning and rarefication of tumor vessels, providing one explanation for reduced tumor growth. Thus, the combination of the two drugs gemcitabine and axitinib has favorable effects on preventing tumor progression in an orthotopic group 3 medulloblastoma xenograft model while exhibiting a favorable toxicity profile. The combination merits further exploration as a new approach to treat high-risk group 3 medulloblastoma.

## Introduction

Medulloblastoma is the most common malignant brain tumor in childhood, requiring intensive multimodal therapy. Next to histology, newly defined molecular subgroups are highly relevant, as revealed by subtype-specific, diverging 5-year-survival rates between 40 and 80%^1,2^. These subgroups, termed WNT-activated group, SHH-activated group, group 3 and group 4, have become part of the new WHO tumor classification^3,4^. Patients with group 3 tumors have the worst prognosis even with the current intensive therapy regimens, consisting of surgery, craniospinal irradiation and, among others, dose-intense cisplatin-, etoposide- and methotrexate-based chemotherapy^5^. Therefore, these patients urgently need additional therapeutic options.

The advent of molecularly defined tumor subgroups sparks great interest in subtype-specific tumor models. In the last few years, several syngeneic mouse models for group 3 medulloblastoma were established to investigate new therapeutic regimens^6–8^. Briefly, essential steps for the tumor model were to overexpress Myc and induce a functional loss of Trp53 in cerebellar cells, leading to highly aggressive medulloblastoma with group 3-properties. By genetically engineering human neural stem and progenitor cells, Hanaford et al. managed to establish a MYC-driven model for group 3 medulloblastoma^9^. A number of xenograft models exists, with the group 3 medulloblastoma cell lines D341, D283, D425 and D458 being used more frequently^10–13^. However, the field is also rapidly evolving towards more patient derived models and databases^14–16^. We recently established and characterized a patient-derived tumor cell line, MB3W1, derived from the pleural effusion of a medulloblastoma with group 3-properties, which had relapsed after chemotherapy and irradiation^17^.

Building on this model, we established a drug panel, including new compounds and focused on substances that are either FDA approved or in the advanced stage of clinical testing, to facilitate rapid clinical translation. As medulloblastoma cells have been described to express VEGF-receptors^18,19^, which may be particularly relevant for group 3 medulloblastoma^19^, the screen included inhibitors of neo-angiogenesis as well as cytotoxic drugs previously identified in an extensive screen in a murine group 3 medulloblastoma model^20^. A combination of the VEGFR-inhibitor axitinib with the nucleoside analogue gemcitabine stood out in terms of in-vitro-toxicity.

Gemcitabine is widely used for several malignant diseases, especially in pancreatic cancer^21^, but experience in medulloblastoma remain scarce. In a phase II study, children with different tumor entities were included at relapse to receive four cycles of gemcitabine and oxaliplatin. Out of 93 evaluable patients, 14 were medulloblastoma patients. One of them achieved a partial response, while six achieved stable disease. The authors concluded that the safety profile was acceptable, although overall the activity in children with relapsed or refractory solid tumors was limited^22^. As a low-toxic regimen, gemcitabine is a palliative option for relapsed patients^23^. As of May 2021, the database clinicaltrial.gov lists three recruiting trials for medulloblastoma patients, which test gemcitabine in various settings and combination therapies (www.clinicaltrials.gov).

Inhibition of angiogenesis in medulloblastoma has been explored using a variety of strategies, but results on efficacy are still inconsistent^24^. VEGF expression is elevated particularly in patients with Group 3 medulloblastoma and is associated with poor survival^19^. Therefore, VEGFR signaling may be a viable target for this tumor type. Axitinib is a small molecular tyrosine kinase inhibitor that selectively inhibits VEGFR-1, -2 and -3 with additionally inhibitory effects against PDGFRβ and c-myc^25^. Although axitinib has been approved for the treatment of therapy-refractory renal cell carcinoma and is being evaluated for other tumor entities in adult oncology, less is known about its effects in medulloblastoma. Recently, in vitro data on axitinib link the drug to growth inhibition and cell death in c-myc-amplified high-risk medulloblastoma, when combined with the PI3K inhibitor GDC-0941^26^.

Here, based upon in-vitro-drug analyses, we identified the combination of gemcitabine and axitinib to be highly cytotoxic for medulloblastoma cells, requiring as little as 30 pM of both drugs to reach half-maximal cytotoxicity, compared to an EC50 of 720 nM for the standard combination of cisplatin and etoposide phosphate. We evaluated this combination in vivo initially in a subcutaneous and ultimately in an orthotopic human group 3 medulloblastoma xenograft mouse model. The drug combination was well-tolerated and showed efficacy in the orthotopic model. In treated mice, 3D light-sheet-fluorescence-microscopy (LSFM) of total tumors revealed thinning and rarefication of tumor vessels, offering one explanation for reduced tumor growth and improved survival. Thus, these two FDA-approved drugs merit further attention as a potential therapeutic approach for high-risk medulloblastoma.

## Results

### MB3W1 medulloblastoma cells express VEGF-receptors and produce VEGF-A

**Figure 1:**
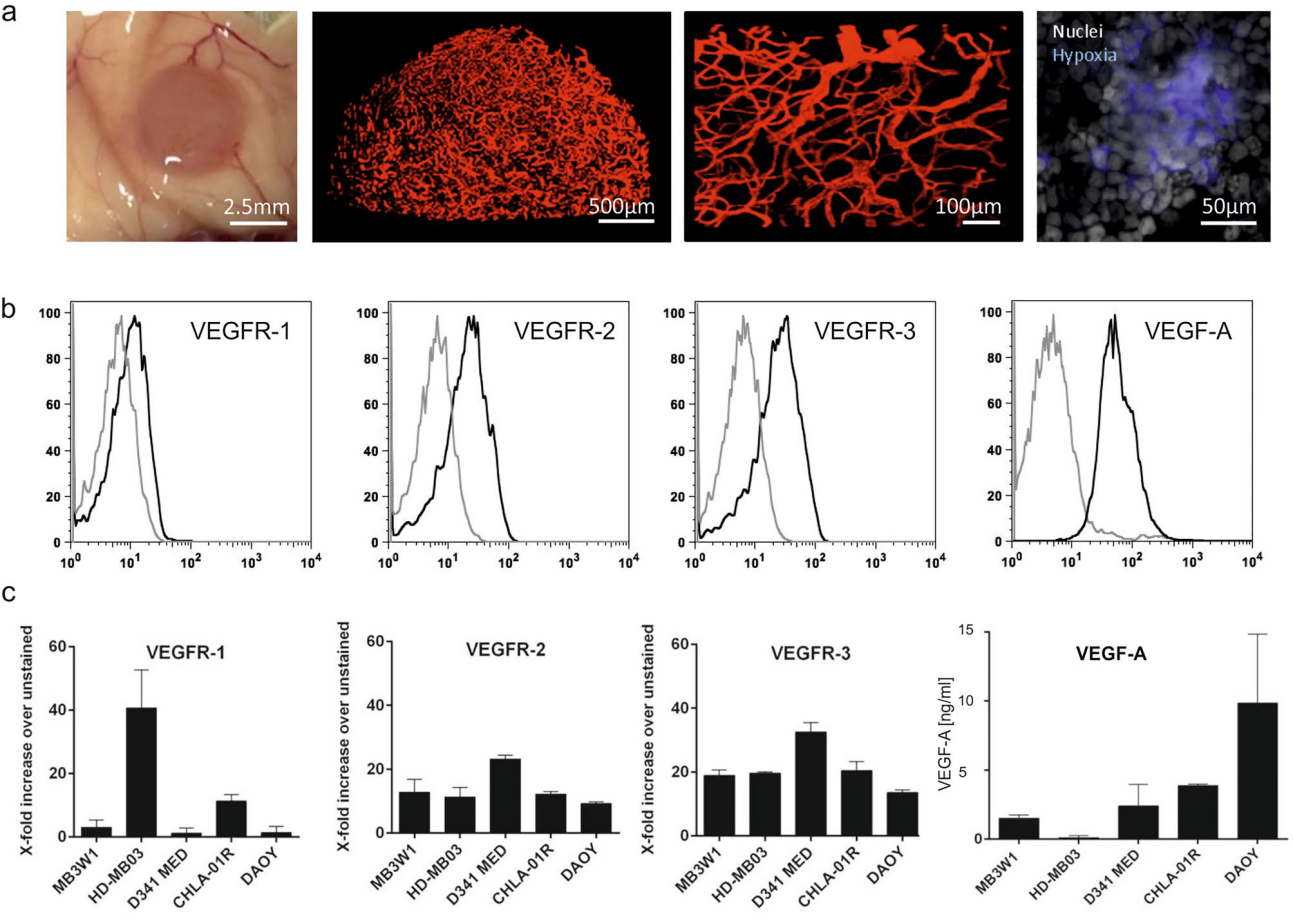
MB3W1 medulloblastoma cells express VEGF-receptors and produce VEGF-A. (**a**) Left: Neo-vascularization of the subcutaneous tumor in NOD/SCID mice. Center left and right: 3D reconstruction of tumor vasculature (VE-Cadherin-AlexaFluor647 staining) with LSFM. Right: MB3W1 are highly hypoxic in the center of spheres as visualized by pimonidazole incubation, which stabilizes thiol-groups in proteins, peptides and aminoacids in hypoxic cells. (**b**) MB3W1 characterization for VEGFR-1, VEGFR-2 and VEGFR-3 expression and VEGF-A production with flow cytometry (black line; grey line: unstained control). (**c**) Expression of VEGFR-1, VEGFR-2 and VEGFR-3 expression of different medulloblastoma cell lines calculated as fold-increase of mean fluorescence intensity (MFI) over the unstained control (mean of 2 experiments). Right panel: VEGF-A concentration as measured with ELISA in supernatants from different medulloblastoma cell lines.

Previously, we established and characterized a patient derived medulloblastoma cell line with group 3 properties^17^. Using DNA methylation-based classification, we confirmed that MB3W1 cells classified, with an optimal calibrated score as Group 3 medulloblastoma (supplemental Fig. 1). Pilot experiments, using a subcutaneous xenograft mouse model, indicated strong neo-vascularization of the tumors and a central hypoxic zone, suggesting a targetable mechanism against tumor growth (Fig. 1a). MB3W1 cells strongly expressed VEGFR-1, VEGFR-2 and VEGFR-3 and produced VEGF-A, which may serve as an endogenous stimulus (Fig. 1b). Expression was comparable to other medulloblastoma cell lines such as DAOY (SHH group), CHLA-01R (group 4), HD-MB03 and D341Med (both group 3) (Fig. 1c)^18,19^.

### In vitro drug screen for single-agent and combinatorial cytotoxicity

**Figure 2:**
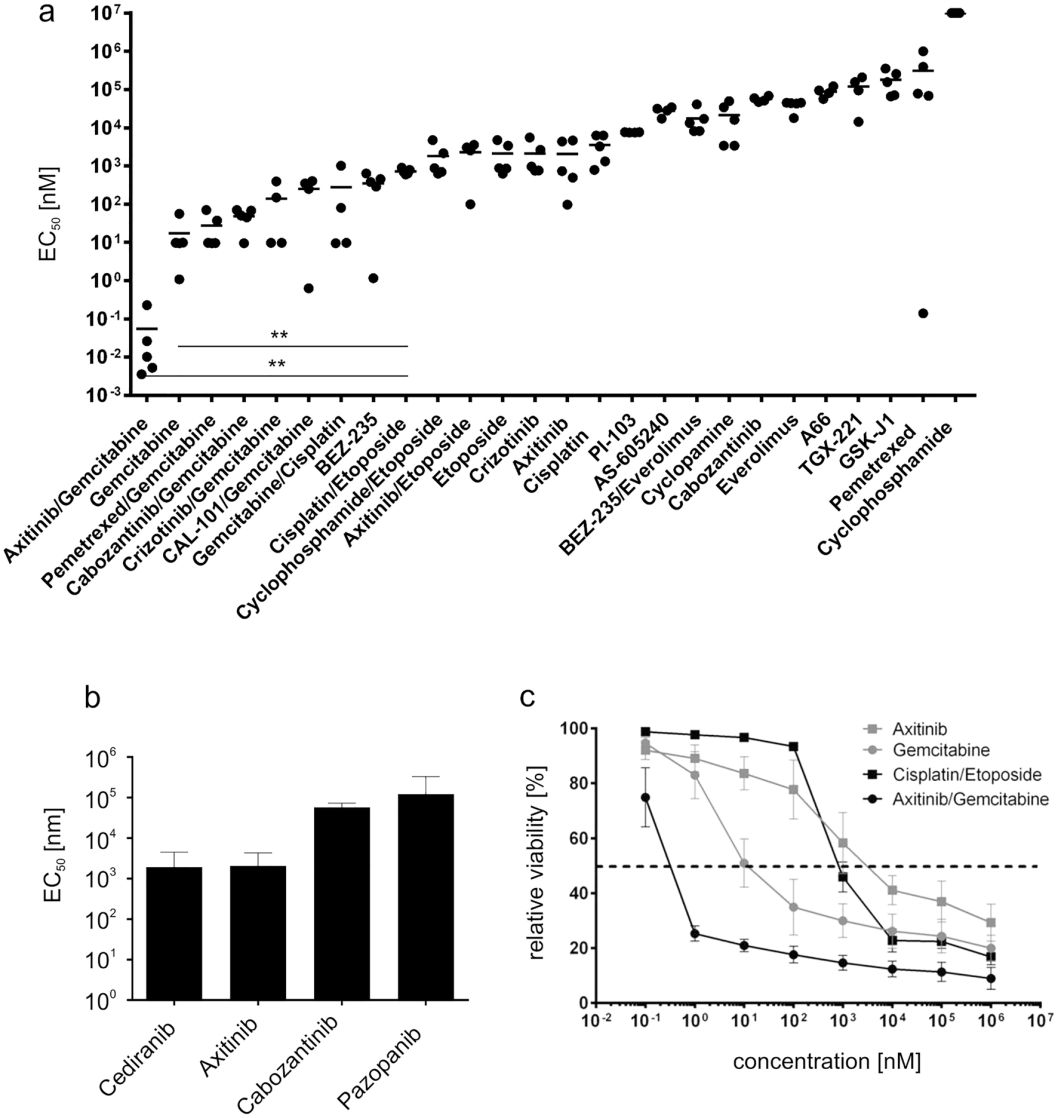
Cytotoxic effect of various cytostatic drugs and inhibitors in vitro. (**a**) MB3W1 cells were treated in vitro with various cytostatic drugs and inhibitors for 48 h before staining with annexin V/Live-Death-staining and flow cytometry analyses. Graph shows the respective EC50 –values (Mean ± SD; combined data from five independent experiments; **, *p* = .0079) (**b**) EC50 –values for a panel of inhibitors of the VEGFR. (**c**) Relative cell viability after cisplatin/etoposide phosphate, gemcitabine, axitinib and gemcitabine/axitinib treatment and corresponding EC50 (dotted line).

Extensive work has been done by Morfouace et al., who identified the combination of gemcitabine and pemetrexed, as effective drug candidates in murine group 3 medulloblastoma cells^20^. Adding to that, we were interested whether targeting VEGFR signaling may affect this tumor entity using a human model. Therefore, we expanded the in vitro screen on approved drugs or drugs in the advanced stage of clinical testing to a total of 24 drugs/inhibitors (VEGRR inhibitors, gemcitabine and others, Fig. 2a and Table 1) in a titrated range from 10 pM to 1 µM. For drug combinations, equimolar concentrations were used and titrated. As reference, we used a combination of cisplatin and etoposide phosphate, as these drugs are the backbone of current clinical chemotherapy protocols^1,5^. After 48 h of drug exposure, we measured annexin-V/LIVE/DEAD cells with flow cytometry and calculated EC_50_ values for each drug or drug combination.

**Table 1:** Drugs and inhibitors used in this screen.

Inhibitor	Target proteins	Clinical use
AS-605240	PI3Kγ	3
Axitinib	VEGFR1, VEGFR2, VEGFR3, PDGFRβ, c-Kit	1
A66	PI3K p110α	3
Bevacizumab	VEGF-A	1
BEZ-235	PI3K p110γ/β/δ, mTOR	3
Cabozantinib	VEGFR2, c-Met, Ret, Klt, Flt1/3/4, Tle2, AXL	2
CAL-101	PI3K p110δ	2
Cediranib	VEGFR, c-Kit, PDGFRβ, CSF-1R, Flt3	2
Cisplatin	DNA-Synthesis	1
Crizotinib	cMET, ALK	1
Cyclopamine	smo (Smothened)-antagonist	2
Cyclophosphamide	alkylating agent	1
Dinaciclib	CDK1, CDK2, CDK5, CDK9	2
Etopophos	Topoisomerase II Inhibitor	1
Everolismus	mTOR	1
Gemcitabine	DNA-analogue	1
GSK-J1	H3K7 Histon-Demethylase	2
GDC-0941	PI3Kα/δ	2
Pazopanib	VEGFR1, VEGFR2, VEGFR3, PDGFR, FGFR, c-Kit, c-Fms	1
Pemetrexed	Antifolat for TS, DHFR and GARFT	1
PI-103	PI3K p110 α/β/γ/δ	3
Quirzatinib	Flt3, KIT, PDGFRα, PDGFRβ, RET, CSF-1R	3
TGX-221	PI3K p110β	3
3-Methyl-adenine	Vps34, PI3Kγ	3

1 = clinically approved, 2 = clinical trial 3 = preclinical testing.

In vitro treatment with any of the VEGF-R inhibitors alone was only effective in the micro-molar range (EC_50_: axitinib (1.5 µM); cediranib (0.5 µM); pazopanib (50 µM); cabozantinib (100 µM)) (Fig. 2b). As a point of reference, half-maximal killing was achieved by 0.8 µM of cisplatin/etoposide phosphate. However, among the eight conditions testing better than cisplatin/etoposide phosphate, seven included gemcitabine as treatment. EC_50_ values reached with a single drug treatment of gemcitabine were as low as 5.8 nM. Although the VEGRR inhibitor alone had cytotoxic effects on MB3W1 cells only in the micro-molar range, effects added to the cytotoxicity of gemcitabine, allowing to titrate the combination as low as 30 pM (Fig. 2a + c). Notably, pemetrexed, the substance gemcitabine was originally combined with^20^, had little effects on MB3W1 cells either alone or in combination with gemcitabine. To explore different cell death pathways, we also evaluated ROS activity and caspase-9 activation for a selected set of drugs. Both pathways were activated in cells treated with gemcitabine and activity peaked in combination with axitinib, but did not reach statistical significance (Supplemental Fig. 2).

**Figure 3:**
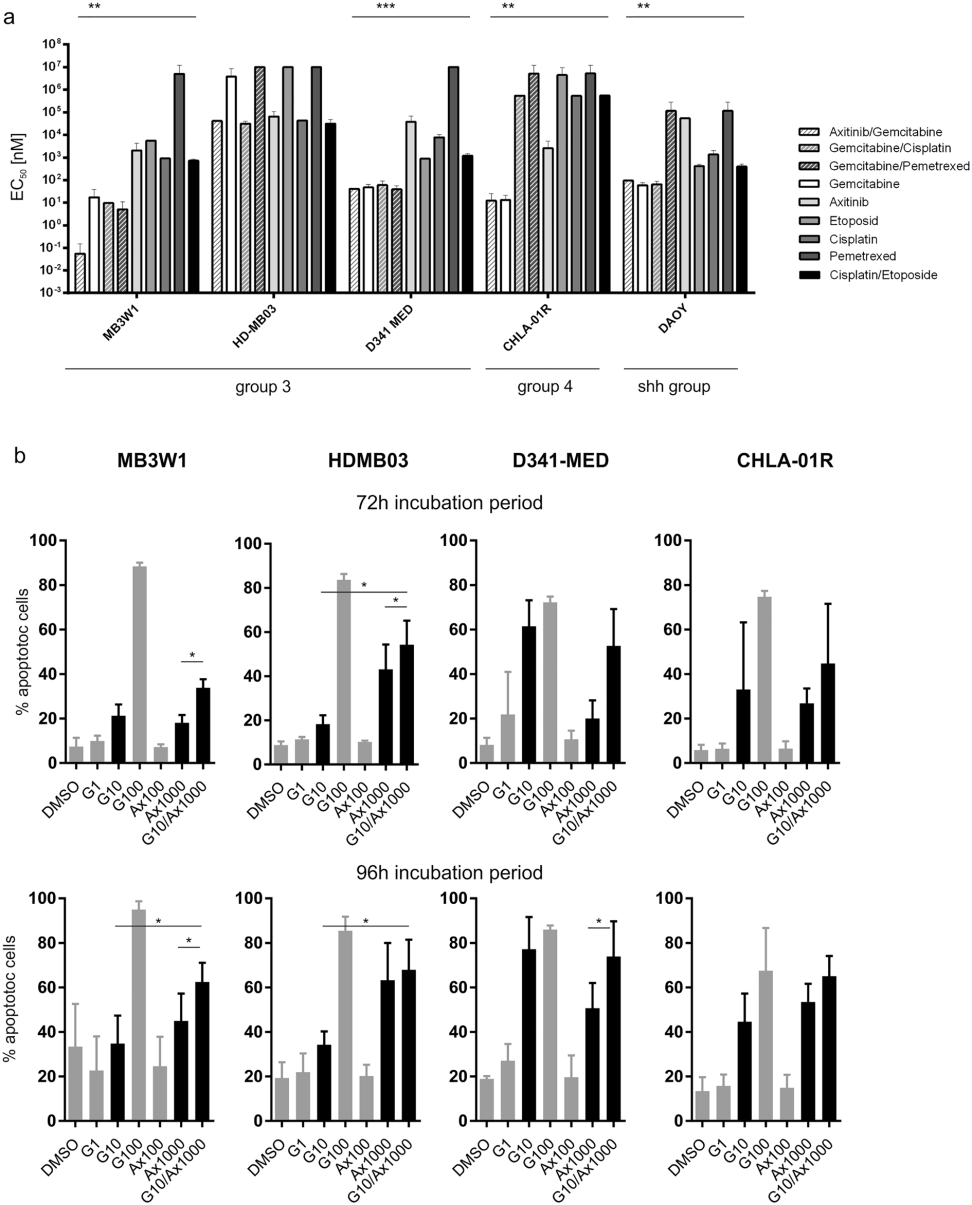
(**a**) EC_50_-values of group 3, group 4 and SHH tumor cell lines treated with different cytostatic drugs (Mean ± SD; representative data from five independent experiments) Gemcitabine/axitinib vs. vehicle: MB3W1: **, *p* = .0079, D341MED: ***, *p* = .0009, CHLA-01R: **, *p* = .0079, DAOY: **, *p* = .005. All experiments involving ‘etoposide’ were done using its phosphate. (**b**) Medulloblastoma cell lines grown in FCS-free conditions and tested for apoptosis 72 h and 96 h respectively using ApotrackerGreen. Cells were exposed to the respective drugs with G1 meaning Gemcitabine 1 nM, other labelling accordingly. Black bars indicate the relevant dosages for mono- and combination therapy. Only this monotherapy was compared to combination therapy (paired t-test; only significant results are shown with *p < .05).Grey bars indicate additional concentrations tested in monotherapy only.

As shown in Fig. 1, HD-MB03 (group 3), D341MED (group 3), CHLA-01R (group 4) and DAOY (SHH group) all expressed VEGFR, lending themselves to VEGF-R inhibition. We therefore asked whether other medulloblastoma cell lines with other molecular subgroups were sensitive to gemcitabine and a combination with axitinib. First, we noted that the EC_50_ for cisplatin/etoposide phosphate as a point of reference varies greatly between the cell lines, with HD-MB03 and CHLA-01R being almost resistant. Gemcitabine alone compared favorably in four of five cell lines tested and was particularly effective for CHLA-01R and MB3W1, the former being rather resistant to all other drugs tested. HD-MB03 showed to be resistant to all of the drugs, requiring high overall drug concentrations. Regarding combination therapy, under these culture conditions, the additional effect by axitinib was only observed in the MB3W1 cells, whereas within the other cell lines the effect in comparison to monotherapy with gemcitabine was similar. (Fig. 3a). Technically, each individual cell line requires different culture conditions and grows differently: Particularly MB3W1 cells are cultured in serum-free conditions, using stem cell medium. The cells grow as spheres with dying cells floating predominantly outside the spheres as single cells. The other cell lines were cultured in medium containing fetal calf serum (FCS), which leads to adherent or at least semi-adherent growth. In order to be able to better compare cytostatic effects, the cells lines HDMB3, D341-MED (group 3) and CHLA-01R (group 4) were gradually weaned off FCS-containing serum and grown in the same FCS-free stem cell medium as MB3W1. The DAOY cell line did not tolerate serum-free conditions. Cells changed their growth behavior, growing non-adherently in spheres similar to MB3W1. We then repeated in vitro assays with these cell lines and assessed the degree of apoptosis after 72 h and 96 h of incubation with either control solvent, gemcitabine, axitinib or the mixture of both. Gemcitabine at a concentration of 100 nM was cytotoxic to all the cell lines, and sub-maximal cytotoxicity was seen at 10 nM , although D341 cells were still very sensitive to monotherapy. HDMB3 responded better than under FCS-conditions, but required a dose of 100 nM. Axitinib in contrast was only active at the micro-molar range, when used as monotherapy in this in vitro setting, as had been established before. However, effects of axitinib on the more resistant cell lines HDMB03 and CHLA-01R were more pronounced under serum-free conditions. For combination treatment in this setting, we chose a suboptimal gemcitabine dose (10 nM) and asked how an optimal axitinib dosing added to cytotoxicity. In this setting, cytotoxic effects of the two drugs were in part additive for HDMB03, which had been shown to be more resistant when cultured in FCS-containing medium. D341-MED cells were particularly sensitive to gemcitabine alone, thus additional axitinib effects could not be demonstrated in the combination treatment. Response to low dose gemcitabine varied greatly in the CHLA-01R cell line (group 4), and an additive effect of the two drugs could not be demonstrated (Fig. 3b). In summary the in vitro analysis showed, that all tumor cell lines were sensitive to low doses of gemcitabine. All cell lines were sensitive to direct effects by axitinib, but only at the micromolar range. Additive effects of both compounds were seen in two of the Group 3 cell lines, when using similar culture conditions.

### Comparison of axitinib to inhibitors with a diverse target spectrum

**Figure 4:**
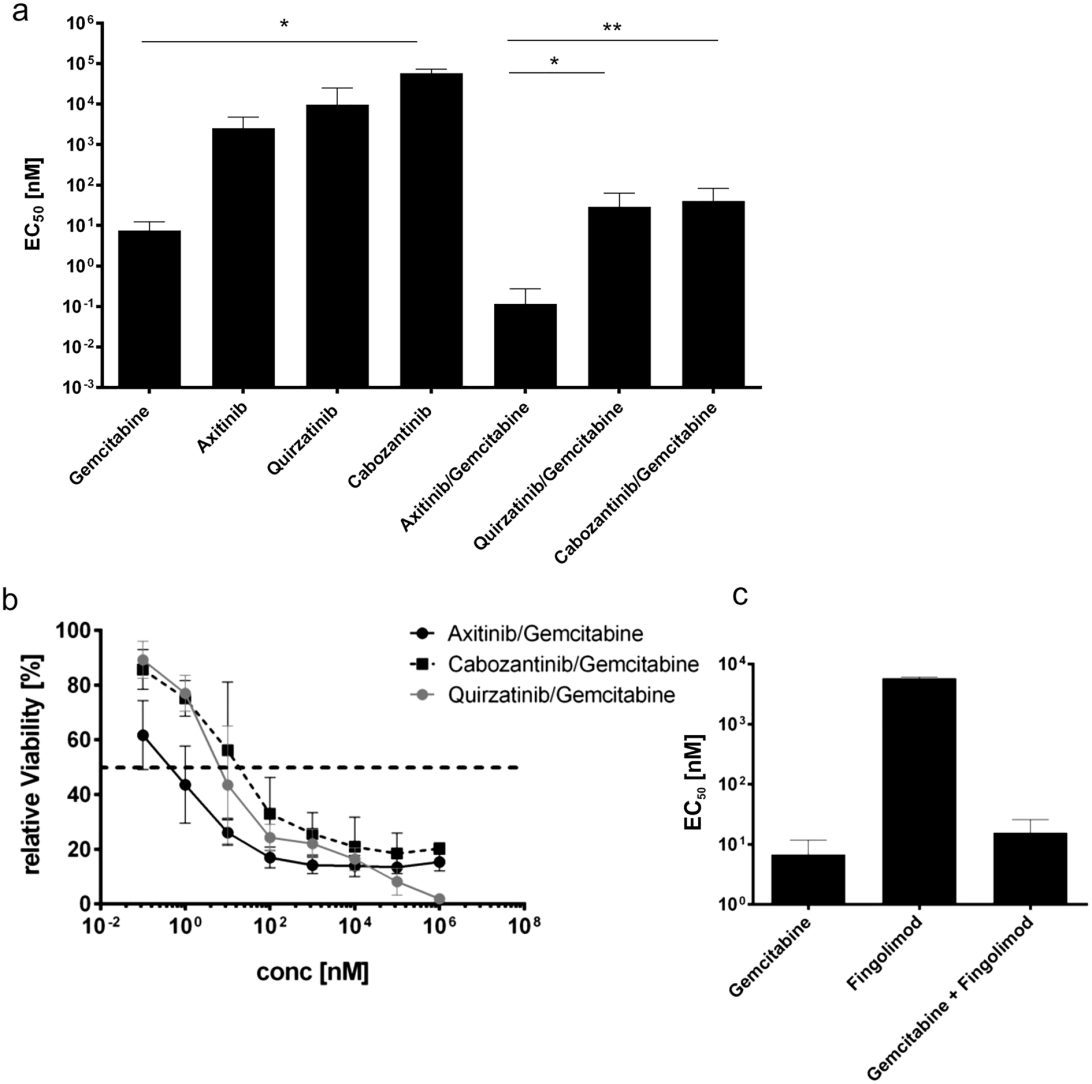
Comparison of axitinib to inhibitors with a diverse target spectrum. (**a**) EC_50_-values of axitinib, quirzatinib (targets: PDGFR, FLT-3, CSF-1R, Kit), cabozantinib (targets: VEGFR-2) treatment and their combination with gemcitabine. Mean ± SD; representative data are from at least two independent experiments. *p < .05, **p < .01. Only single drugs to each other or drug combinations to each other were compared. (**b**) Relative cell viability after axitinib/gemcitabine, cabozantinib/gemcitabine and quirzatinib/gemcitabine treatment and respective EC_50_-values (dotted line). (**c**) EC_50_-values of fingolimod treatment and the combination with gemcitabine.

Axitinib is not only a pan-VEGFR-inhibitor but also targets PDGF-receptors (PDGFR) and c-Kit^25^. To evaluate to which extent such effects contribute in the observed cytotoxicity in MB3W1 cells, we selected inhibitors with an overlapping target spectrum but lacking VEGFR-inhibition. Quirzatinib is an inhibitor of FLT3, PDGFR, Kit and CSF-1R, thus sharing multiple targets with axitinib but lacking activity against VEGFR. When tested in MB3W1 cells, quirzatinib alone had a tenfold higher EC_50_ than axitinib alone (Fig. 4a) and did not show any additive effects in combination with gemcitabine. This may indicate that inhibition of PDGF or Kit signaling is not the main contributing mechanism to explain the cytotoxic effects seen with the gemcitabine/axitinib combination. Furthermore, additional testing of a more selective VEGFR-2-inhibitor, cabozantinib, in combination with gemcitabine did not increase cytotoxic activity, stressing that high selectivity may not be of advantage once multiple, redundant pathways are activated (Fig. 4b). To further explore the potential mechanisms of the additive effects observed by axitinib, we also asked, whether blockade of the S1PR1/STAT3 loop using the Sphingosine 1-phosphate receptor 1 antagonist FTY720 (Fingolimod) would bear similar results. Few reports point towards STAT3 signaling as a potential target for axitinib, rendering tumor cells more sensitive to gemcitabine^26–28^. In this context, blockade of the Spingosine 1 phosphate receptor 1/STAT3 loop using the S1PR1 antagonist FTY720 led to decreased tumorigenesis in group 3 medulloblastoma patient-derived xenografts^29^. However, in our in vitro assays, FTY720 (Fingolimod) did not show any clear additive effects, when combined with gemcitabine (Fig. 4c).

### Axitinib and gemcitabine treatment in a subcutaneous tumor model

**Figure 5:**
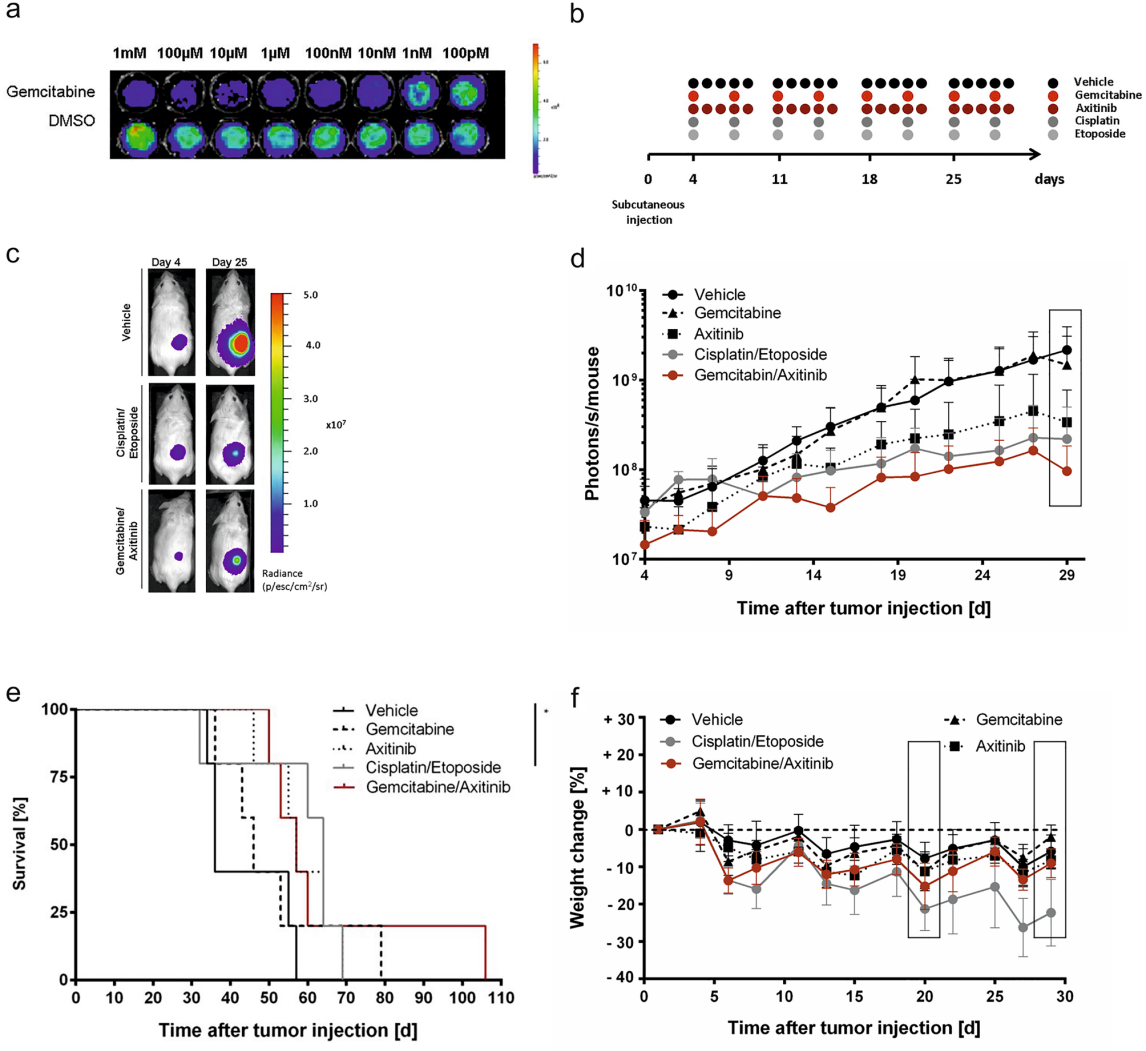
Axitinib and gemcitabine treatment in a subcutaneous xenograft tumor model. (**a**) Representative in vitro bioluminescence signal of MB3W1 after 48 h treatment with gemcitabine or vehicle control. (**b**) Experimental strategy: Injection of 1 × 10^6^ MB3W1 in the right flank of NOD/SCID mice on day 0. On day 4 treatment of mice with cytostatic drugs started once weekly (gemcitabine, cisplatin, etoposide phosphate) and 5 times per week (axitinib), respectively, in 4 cycles. Tumor growth was monitored with non-invasive BLI. (**c**) Representative pictures of the BLI-Signal at the beginning (d4) and during therapy (d25). (**d**) Quantitative analysis of BLI-signals in different treatment groups (n = 5); data displayed as mean ± SD. Two-way ANOVA for multiple comparisons was perfromed on day + 29 (box) with the following p values: Cisplatin/Etoposide vs vehicle: ****; Gemcitabine/Axitinib vs vehicle: ****; Axitinib vs vehicle: ****; Gemcitabine vs vehicle: n.s.; Cisplatin/Etoposide vs Gemcitabine: **; Axitinib vs Gemcitabine: **; Gemcitabine/Axititinib vs Gemcitabine: ***. (**e**) Survival curves of treatment groups. Cisplatin/Etoposide vs Vehicle: *; all other comparisons: n.s. (**f**) Comparison of weight change as an indicator of toxicity. Two-way ANOVA for multiple comparisons was done on two specific time points (boxes) and a significant difference was indicated as follows: day + 20: Cisplatin/Etoposide vs Vehicle: ****; Cisplatin/Etoposide vs Gemcitabine: **; Cisplatin/Etoposide vs Axitinib: **; all other comparisons: n.s.; day + 29: Vehicle vs Cisplatin/Etoposide: ****; Cisplatin/Etoposide vs Gemcitabine: ****; Cisplatin/Etoposide vs Axitinib: ****; Cisplatin/Etoposide vs Gemcitabine/Axitinib: ***; all other comparisons: n.s.

In light of our in vitro findings and the fact that the combination of gemcitabine and axitinib is being evaluated for other tumor entities^30^, we were interested to pursue combination therapy with gemcitabine and axitinib in the xenograft model. To assess drug activity in vivo, MB3W1 cells were stably transduced with luciferase and enhanced Green Fluorescent Protein (eGFP). Transduced cells showed similar growth kinetics as untransduced cells (not shown) and responded similarly to gemcitabine in vitro (Fig. 5a). Initial testing was performed in a subcutaneous tumor model. MB3W1 cells (10^6^) were injected subcutaneously into NOD/SCID mice. After 4 days, when tumors were established, mice were treated either with gemcitabine or cisplatin and etoposide phosphate intraperitoneally (i.p.) for four weeks. Mice, which belonged to gemcitabine/axitinib group were treated once a week with gemcitabine and five times per week orally (p.o.) with axitinib for four weeks. Furthermore, one group included mice receiving axitinib orally as monotherapy (Fig. 5b). Tumor growth was monitored by BLI and health status of mice was monitored by inspection and weight analysis. In this setting, cisplatin and etoposide phosphate, gemcitabine and axitinib and axitinib-only treated mice, respectively, caused reduced tumor growth of MB3W1 in comparison to vehicle, whereas gemcitabine monotherapy did not. This started to be statistically significant by day 25 until the end of the experiment on day 29 (Two-way ANOVA, multiple comparisons). Comparing the two respective combinations with each other showed no difference, suggesting similar effectivity in the subcutaneous model (Fig. 5c + d). Evaluating survival, only the cisplatin/etoposide phosphate group reached the level of a significant survival advantage (*p* = 0.035), although the curve suggests a positive impact of gemcitabine/axitinib treatment as well (Fig. 5e). In terms of toxicity, axitinib/gemcitabine-treated mice tolerated the therapy better than the cisplatin/etoposide phosphate treated mice, as shown by clinical signs of distress (fur ruffling) and weight loss. Two-way ANOVA for multiple comparisons showed a very clear difference between the cisplatin/etoposide phosphate vs. the gemcitabine/axitinib-group (and monotherapy vs. either axitinib or gemcitabine). In contrast, comparison between gemcitabine/axitinib and vehicle showed no difference, underscoring that this treatment option caused no major toxicity (Fig. 5f).

### Axitinib and gemcitabine treatment in the intracranial tumor model

Next, we tested this drug combination in the intracranial medulloblastoma model, mimicking a clinically more relevant environment for the tumor cells. Previous studies had shown that as little as 5 × 10^3^ MB3W1 cells injected orthotopically into the right hemisphere of the cerebellum were sufficient to form tumors recapitulating the original phenotype^17^. Tumor cells were allowed to establish themselves for 11 days in the cerebellum before treatment was started and monitored with non-invasive BLI. As before, we applied cytostatic drugs i.p. once a week (gemcitabine, cisplatin, etoposide phosphate) and axitinib p.o. daily for four weeks.

**Figure 6:**
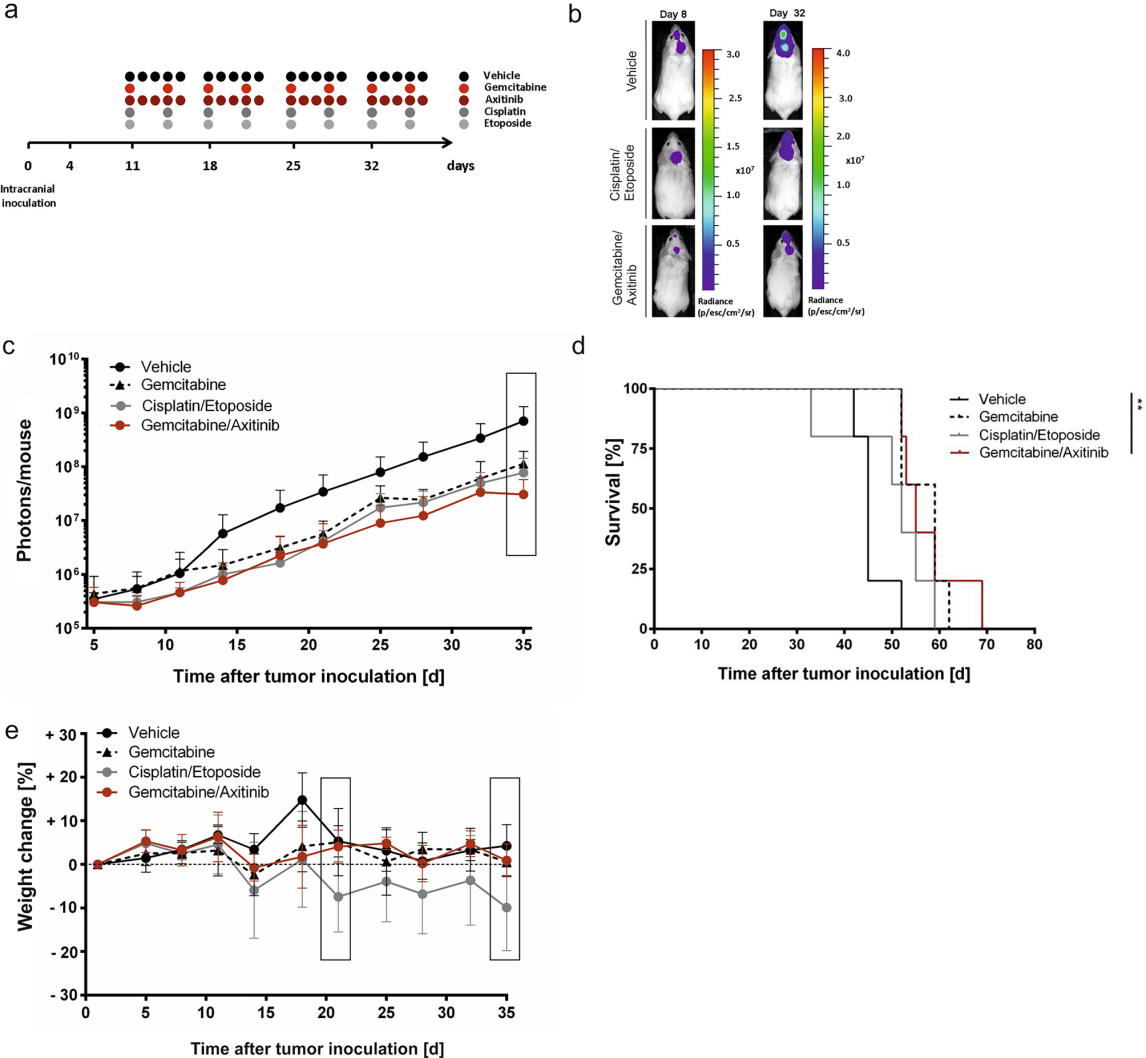
Treatment comparison in the intracranial medulloblastoma model. (**a**) Experimental strategy: Injection of 5 × 10^4^ MB3W1 group 3 medulloblastoma cells into the right hemisphere of the cerebellum of NOD/SCID mice. Starting on day 11 after tumor inoculation, mice were treated with cytostatic drugs once weekly (gemcitabine, cisplatin, etoposide phosphate), and 5 times per week (axitinib), respectively, in 4 cycles. Tumor growth was monitored with non-invasive BLI. (**b**) Representative pictures of the BLI-signal at the beginning (d11) and during therapy (d32). (**c**) Quantitative analysis of in vivo BLI-signals in different treatment groups (n = 5); data displayed as mean ± SD, Two-way ANOVA for multiple comparisons were performed and significant p-values for d35 (box) were as follows; Cisplatin/Etoposide vs Vehicle: ****; Gemcitabine/Axitinib vs Vehicle: ****; Gemcitabine vs Vehicle: ****. (**d**) Survival curves for the respective treatments. Gemcitabine/Axitinib vs Vehicle: **; all other comparisons: n.s. (**e**): Mean of weight change to detect toxicity. Two-way ANOVA for multiple comparisons was done on two specific time points (boxes) and a significant difference was indicated as follows: day + 21: Cisplatin/Etoposide vs Vehicle: **; Cisplatin/Etoposide vs Gemcitabine: **; Cisplatin/Etoposide vs Gemcitabine/Axitinib: **; all other comparisons: n.s.; day + 35: Cisplatin/Etoposide vs Vehicle: ***; Cisplatin/Etoposide vs Gemcitabine: *; Cisplatin/Etoposide vs Gemcitabine/Axitinib: *; all other comparisons: n.s.

Compared to the vehicle group, BLI imaging showed a significantly reduced tumor growth for both treatment combinations and, in this setting, also for the gemcitabine monotherapy. Based on previous in vitro and in vivo data and in observance of the reduction principle, axitinib monotherapy was not evaluated in the orthotopic setting (Fig. 6a–c). Delay in tumor growth translated into a survival advantage when compared to the vehicle control group, which reached statistical significance only for the gemcitabine/axitinib treated animals (Fig. 6d). Direct comparison of different treatments with each other did not reach statistical significance.

As observed before in the subcutaneous model, mice tolerated gemcitabine/axitinib significantly better than cisplatin/etoposide phosphate regarding weight loss and fur ruffling. While the gemcitabine/axitinib treatment did not cause any significant weight change compare to vehicle controls, cisplatin/etoposide phosphate clearly did, with differences towards vehicle or the gemcitabine/axitinib group (Fig. 6e).

We conclude that in the orthotopic model, gemcitabine, either alone or in combination with axitinib, showed a comparable activity against the tumor, while being better tolerated than the standard treatment with cisplatin/etoposide phosphate.

### Quantitative analysis of angiogenesis in orthotopic group 3 medulloblastoma with 3D-LSFM

**Figure 7:**
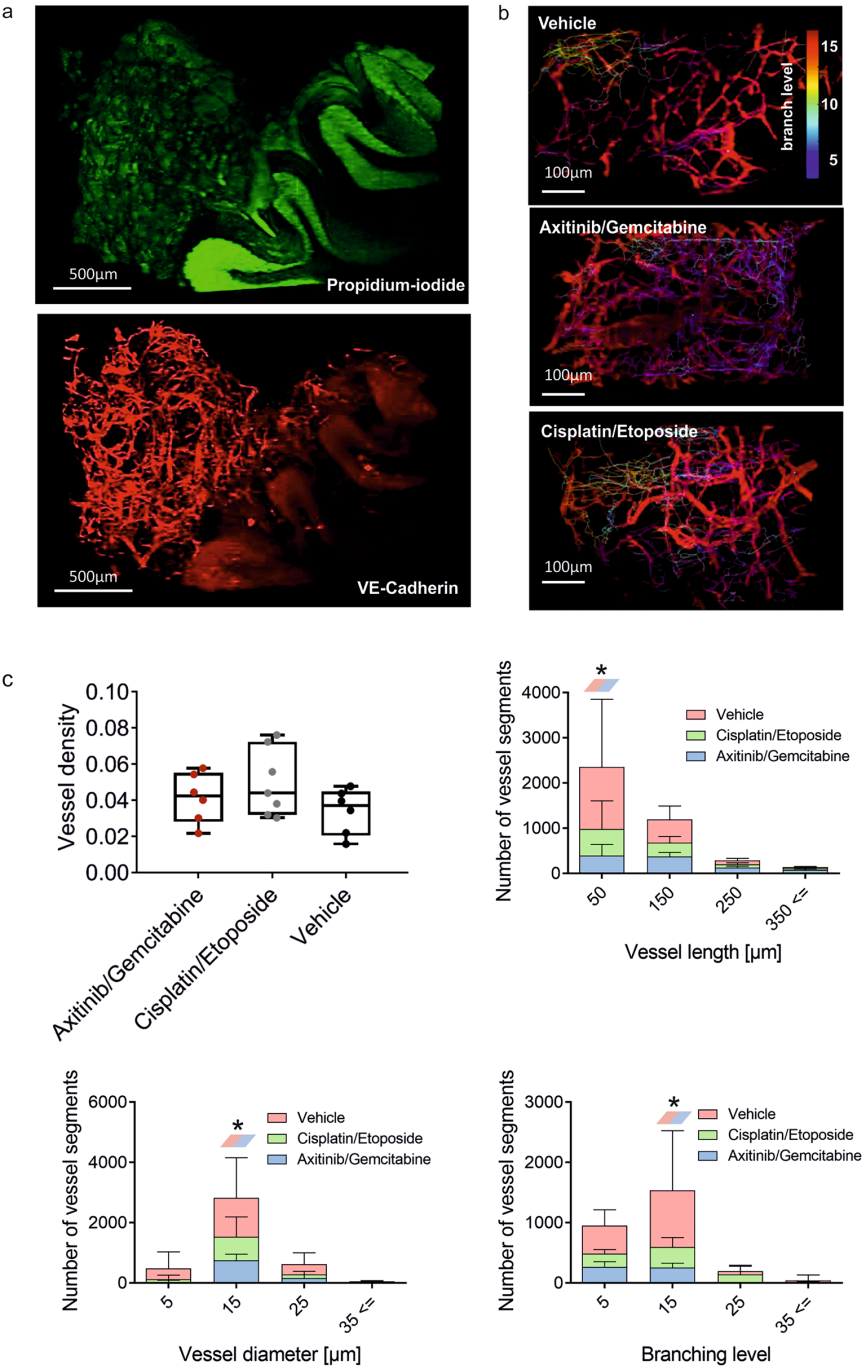
Blood vessel visualization and quantification in an orthotopic group 3 medulloblastoma with 3D-LSFM. At the endpoint of the experiment, blood vessels were stained in vivo with anti-VE-Cadherin Alexa Fluor 647, 30 min before transcardial perfusion of mice. Stained and optically cleared brain samples were imaged with a home-built LSFM. (**a**) 3D reconstruction of orthotopic medulloblastoma in the murine cerebellum after detection of propidium iodide (green) and VE-Cadherin (red) with a 5 × objective (scale bar: 500 µm). (**b**) Blood vessel structure and filament analyses of vasculature in orthotopic tumors after different treatment regimens (scale bar: 100 µm). Vasculature segments are color coded based on branch level. (**c**) Quantitative analysis of overall blood vessels density, vessel length, diameter and branch level distribution under different treatment conditions. Bar graphs present mean ± SD, n = 3, vessel length: Gemcitabine/Axitinib vs vehicle, *p = .047, vessel diameter: Gemcitabine/Axitinib vs vehicle, *p = .042, branching level: Gemcitabine/Axitinib vs vehicle, **p = .009 (Two-way-ANOVA).

We next addressed how this combination therapy would affect tumor morphology and, more specifically, vascular architecture. As a method of choice, we employed 3D light-sheet fluorescence microscopy (LSFM). This new technology allows to optically scan intact tumors including the adjacent tumor microenvironment and to quantify treatment effects on blood vessel formation and vascular density^31,32^. Evaluating the orthotopic model (Fig. 7a and b), the analysis of overall vessel density did not reveal any significant differences between the treatment groups (Fig. 7c, upper left). However, when evaluating the vessel architecture in more depth, untreated (vehicle) tumors showed a significantly higher number of shorter vessel segments (*p* = 0.047), also differing in vessel diameter (*p* = 0.042) and branching level (*p* = 0.009), when compared to the axitinib/gemcitabine group, which was not seen in the cisplatin/etoposide phosphate group (Fig. 7c).

## Discussion

There is an unmet medical need for better treatment options in high-risk medulloblastoma with a molecularly unfavorable subtype. Dose-intensive chemotherapy is already part of current therapy protocols, precluding dose-intensification. Clearly, additional treatment strategies with a favorable toxicity profile are urgently needed.

Here, we confirm previous pre-clinical findings, that gemcitabine, which is currently not part of the first-line treatment strategy in international protocols, is active in Group 3 medulloblastoma cells^20^. Moreover, we found indications that combination therapy with axitinib may be advantageous, adding direct and indirect effects by VEGFR-inhibition, thereby reducing the required dose for gemcitabine and thus increasing tolerability. This combination emerged from an in vitro screen of substances, which was designed to include approved drugs or drugs in the advanced stage of development. Inspired by the extensive screen performed by Morfouace et al.^20^ in murine medulloblastoma, the most promising candidates in their screen, pemetrexed and gemcitabine, were included in our study. Testing drugs in vitro first, gemcitabine,—but not pemetrexed -, was confirmed to be highly effective. In this test system, axitinib in combination with gemcitabine increased cytotoxicity. When evaluated in the subcutaneous and orthotopic animal setting, this combination slowed down tumor growth to a similar degree as the therapy with cisplatin/etoposide phosphate and was better tolerated in terms of toxicity. As the EC_50_ particularly for gemcitabine was several logs lower than for cisplatin/etoposide phosphate (and other combinations), one can expect that clinically relevant serum levels can be achieved in the clinical setting.

The subcutaneous xenograft model allowed us to evaluate gemcitabine and axitinib separately as monotherapy, and surprisingly gemcitabine monotherapy did not affect tumor growth, whereas axitinib monotherapy in this model showed significant tumor growth impairment. The survival curve suggests, that axitinib has additive effects, as only the combination therapy axitinib/gemcitabine reached statistical significance, whereas gemcitabine monotherapy did not. Interestingly in the aforementioned, recently published paper for screening new drugs^16^ using several prediction algorithms on PDX models, axitinib turned out to be among the top 25 substances tested, using gene expression data and the DiSCoVER algorithm^9^ to generate predictions of drug sensitivity. Axitinib there ranked higher than, e.g. vorinostat or even cisplatin. However, additional confirmatory assays, including monotherapy with each of the drugs as well as other models are important future steps.

The dosages used in these experiments, were based on other publications as well as preliminary dose finding experiments to avoid excess toxicity. Comparing dosage per kg and comparing it to protocols in humans, shows that the dose for cisplatin and etoposide phosphate was in the higher range to that used in humans, whereas gemcitabine may even have been slightly under-dosed. Even, if such direct comparison between mice and humans can only allow a rough estimate, the very same drug combination has already been evaluated in adults: a phase III trial for patients suffering from metastatic or advanced pancreatic carcinoma showed no benefit of adding axitinib to gemcitabine in this particular patient cohort, but also demonstrated an acceptable toxicity profile^30^. Likewise, a phase II study on non-small-cell lung cancer adding axitinib to gemcitabine and cisplatin considered the combination to be safe, whereas the study design precluded any conclusion regarding efficacy^33^. We conclude that this drug combination has an acceptable—and in comparison to other intensive chemotherapy even favorable—toxicity profile, which therefore would not impede clinical testing in group 3 medulloblastoma patients.

When we evaluated other medulloblastoma cell lines, we noticed the wide range in sensitivity. HD-MB03 cells were almost resistant to all drugs tested, when performing the assays in FCS-containing medium. This somewhat changed, when the cell lines were weaned off of FCS and cultured in serum-free stem cell medium, just like MB3W1. Growth characteristics visibly changed, as cells grew in non-adherent spheres. The cells were now sensitive to gemcitabine monotherapy in the range of 10-100 nM, and axitinib had an additive effect. The other group 3 cell line tested, D341-MED, proved extremely sensitive to gemcitabine alone (about 80% cell death at 10 nM), therefore a clear additional effect of axitinib could not be observed. Although all three cell lines are characterized as group 3 medulloblastoma, as confirmed by our own methylation data (Suppl. Fig. 1), there certainly are individual differences in chemo-sensitivity, that cannot be explained by one single factor. There are differences in the origin of the cells (pleural effusion of metastatic cells (MB3W1) vs. original primary tumor (HDMB03)), but also a different history regarding culture conditions (the MB3W1 cells have never been cultured in serum-containing medium). Further studies, particularly testing various freshly isolated tumor samples in PDX models, will help to assess how beneficial a combination therapy might be and whether the effects on vessel length and thickness, observed in this orthotopic model, are a true consequence of axitinib mediated anti-angiogenic indirect effects in the in vivo setting.

As demonstrated by Thompson EM et al., VEGF expression is elevated particularly in patients with Group 3 medulloblastoma and is associated with survival^19^. On condition of stratification according the molecular subgroup 3, the authors suggest that anti-angiogenic therapy may in fact be useful for these patients. Data on the medulloblastoma microenvironment, not only at the time of diagnosis, but also throughout therapy and at time of relapse are still scarce. A mass spectrometry-based study of cerebrospinal fluid (CSF) from recurrent medulloblastoma patients, makes the case of a tumor-promoting, anti-inflammatory and hypoxic environment, supporting drug resistance and stem cell properties^34^.

Of note, in our approach, we started therapy once tumors were established, 4 and 11 days after tumor engraftment, respectively. Such delayed therapy regimen may better translate to the clinical situation of pre-established tumors in patients with a status of minimal residual disease e.g. after initial surgery. Especially for medulloblastoma, the clinician encounters varying scenarios regarding the tumor load: e.g., patients undergoing complete resection may still be CSF positive for tumor cells (M1) and may benefit from swift, less-toxic chemotherapeutic treatment. This is the situation where current chemotherapeutic strategies still require a significant time delay to allow wound healing, potentially favoring growth of metastatic tumor cells. This critical period could potentially be shortened using a less toxic, equally effective therapy option. A less toxic treatment strategy is also needed in the relapse situation, as patients are heavily pretreated and repeating dose intensive chemotherapy cycles would neither be tolerated nor be useful.

In summary, our data confirm that gemcitabine exerts favorable cytotoxic activity on medulloblastoma cells. Combination with axitinib increases these effects, while at the same time the combination therapy shows favorable tolerability in the xenograft model. This data suggests that this combination merits further exploration as a new approach to treat aggressive group 3 medulloblastoma.

## Materials and methods

### Tumor cell lines

The human group 3 medulloblastoma tumor cell line MB3W1 was cultured in DMEM/F12 (Thermo Scientific, Waltham, MA, USA) supplemented with 20 ng/ml basic fibroblast growth (bFGF, Peprotech), 20 ng/ml epidermal growth factor (EGF, Peprotech, Hamburg, Germany), 2% B-27 supplement (Thermo Scientific), 1% MEM Vitamins (Thermo Scientific), 40U/ml penicillin (PAA) and 40 µg/ml streptomycin (PAA). HD-MB03 (Group 3) was purchased from the German Collection of Cell Cultures (DSMZ, Braunschweig, Germany) and was maintained in culture with RPMI 1640 medium (Thermo Scientific) containing 10% FCS (Thermo Scientific) and 25 IU/ml Penicillin + 25 IU/ml Streptomycin. D341 MED (Group 3) and CHLA-01R (Group 4) were purchased from the American Type Culture Collection (ATCC, Virginia, USA). D341 MED was cultured in αMEM (Thermo Scientific) containing 20% FCS, 25 IU/ml Penicillin + 25 IU/ml Streptomycin. CHLA-01R was cultured in DMEM/F12 containing βFGF, EGF, B-27 supplement and MEM vitamins. DAOY (SHH) was kindly provided by C. Hagemann (Dept. of Neurosurgery, University Hospital Würzburg) and was cultured in DMEM (Thermo Scientific) containing 10% FCS, 25 IU/ml Penicillin + 25 IU/ml Streptomycin.

### Cytostatic drugs and inhibitors

Cisplatin, gemcitabine, pemetrexed and etoposide phosphate (etopophos) in its dissolved form were obtained from the pharmacy of Würzburg University Hospital. All other inhibitors and cytostatic drugs were purchased from Selleckchem (Houston, TX, USA). Inhibitors were diluted in DMSO for in vitro experiments following the instructions of the manufacturer. Axitinib was solved in 0.5% carboxymethylcellulose (Merck, Darmstadt, Germany) for mouse experiments. Vehicle controls were performed using the respective solvent.

### Cytotoxicity assays

Tumor cells were cultured with a density of 5 × 10^5^ cells/well in 96-well plates for 48 h adding various cytostatic drugs and inhibitors at different concentrations (10^–1^ to 10^7^ nM), alone and in combinations, before staining with annexin V-APC (Biolegend, San Diego, CA, USA) and LIVE/DEAD fixable death cell stain kit (Thermo Scientific) and analyzing by flow cytometry. Survival rates for treated groups were normalized to the survival rate of the vehicle group to generate survival inhibition curves and EC_50_ values for individual cytostatic drugs^35^. Additional experiments with cell lines adapted to FCS-free stem cell medium were performed by staining apoptotic cells with Apotracker-Green (Biolegend).

### Flow cytometry

Tumor cells were resuspended in Phospate-buffered saline (PBS) (PAN Biotech, Aidenbach, Germany) including 1% FCS before staining with antibodies for 20 min at room temperature. For intracellular staining, cells were fixed and permeabilized by using Permeabilization Buffer and Fixation/Perm Concentrate (Thermo Scientific). Anti-CD304-AlexaFluor647 (12C2), anti-CD309-AlexaFluor647 (7D4-3), anti-VEGF-A-APC (polyclonal) and anti-VEGFR-3-APC (9D9F9) antibodies were obtained from Biolegend. Anti-caspase-9-unconjugated antibody (polyclonal) was obtained from Cell Signaling Technology (Leiden, Netherlands) and anti-VEGF-C-Biotin (polylconal) was obtained from Abcam (Cambridge, MA, USA). Anti-VEGFR-1-APC (40,560) was obtained von R&D Systems (Mineapolis, MN, USA). Streptavidin-546 (Thermo Scietific) was used as a secondary staining reagent. For reactive oxygen species (ROS) detection, tumor cells were labeled with ROS-working solution (Abcam) for 1 h at 37 °C, before incubation with cytostatic drugs and inhibitors for 24 h at 37 °C. All measurements were performed on a FACS Canto II (BD, Heidelberg, Germany) and were analyzed with FlowJo Software (Tree Star, Ashland, OR, USA).

All animal experiments were approved by the Committee on Animal Experimentation of the Regierung von Unterfranken as the responsible authority (Permit Number 55.2-DMS-2532–2-42) and complied with German animal protection law. The study is reported in accordance with ARRIVE guidelines (https://arriveguidelines.org). Female NOD.CB17-Prkdcscid/J (NOD/SCID), 8–12 weeks old, were purchased from Jackson Laboratory and housed under specific pathogen free conditions. For acclimatization, mice were housed in the local animal facility for at least one week before the start of the experiment. For subcutaneous tumor studies, mice were injected in the right flank with 1 × 10^6^ lentivirally transduced MB3W1 that stably expresses firefly luciferase (FLuc) and eGFP). Tumor cells were allowed to engraft for 4 days. Subsequently, mice were treated either with gemcitabine (30 mg/kg) or cisplatin (5 mg/kg) and etoposide phosphate (etopophos 15 mg/kg) by weekly i.p. injections for four weeks. Mice, which belonged to the gemcitabine/axitinib group were treated once a week with gemcitabine (30 mg/kg i.p.) and five times per week p.o. with axitinib (25 mg/kg) for four weeks. Drug doses were based on previously published protocols and preliminary dose findings experiments. In the orthotopic medulloblastoma model, 5 × 10^4^ tumor cells were inoculated in the right hemisphere of the cerebellum (2 mm lateral, 2 mm caudal of lambda) by employing a stereotaxic frame as previously described^36^. Here, tumors were allowed to establish themselves for 11 days. Then, mice were treated under the same conditions as in the subcutaneous model for four weeks. Subsequently, health status, body weight and tumor growth with BLI were checked every 2–3 days. Animals were euthanized when they showed first symptoms of disease or lost 20% of their body weight.

### In vivo BLI

Mice were injected i.p. with a mixture of Ursotamin (50 µg/g, Serumwerk, Bernburg, Germany), Xylavet (5 µg/g, CP-Pharma, Burgdorf, Germany) and D-Luciferin (300 mg/kg, Biosynth, Sankt Gallen, Switzerland). After 10 min of incubation, imaging of the mice was performed with an IVIS spectrum imaging system (Perkin Elmer, Waltham, MA, USA)^23^. Data were analyzed with Living Image 4.0 (Perkin Elmer) and Prism 6 software (GraphPad, San Diego, CA, USA).

### Immunofluorescence staining

For analyzing hypoxic cells, tumor cells were incubated for 1.5 h with 150 µM Pimonidazol at 37 °C. Pimonidazol is only active in hypoxic cells and stabilizes covalent bound thiolgroups in proteins, peptides and amino acids. After preparing cytospins, tumor cells were stained with secondary Hypoxyprobe-RedAPC antibody over night at 4 °C. Slides were washed with PBS and mounted with Vectashield mounting medium including DAPI (Vector laboratories, Burlingame, CA, USA).

### Light-sheet-fluorescence-microscopy (LSFM)

For LSFM analyzes mice were sacrificed after four weeks of therapy on day 36. 2 h after injection of 20 µg VE-Cadherin-AlexaFluor647 intravenously, mice were anesthetized and transcardially perfused with 4% paraformaldehyde (PFA) (Carl Roth, Karlsruhe, Germany) and the brain was prepared and fixed with 4% PFA for 2 h. Samples were blocked with 2% FCS/PBS in 0.1% Triton-X (Carl Roth), incubated with propidium iodide (1 µg/ml, Merck) for 60 min at 4 °C. Organs became optically transparent after dehydration in a graded ethanol series (30–100% ethanol, each 1.5 h), followed by a clearing procedure including 2 h in 100% n-hexane (Merck) and 3 × 30 min clearing solution containing 1 part benzyl alcohol (Merck) and 2 parts benzyl benzoate (Merck). For 3D-imaging, we employed a homebuilt LSFM as previous described^37^. The resulting multicolor stacks were processed and analyzed with Imaris Software 7.7.2 (Bitplane, Zurich, Switzerland). 3D reconstruction of vasculature, vasculature segmentation and analysis were performed with Imaris 8.1 software. Diameter, length as well as branch level were calculated for each dendrite of each vessel and the results were presented as histograms. Branch level was determined according to the new branching point and diameter change of segments. The branch level increased as soon as the segment diameter decreased in the branching point.

### Statistics

Prism 6.0 software was used for statistical analyses and p values smaller than 0.05 were deemed significant. Significance was calculated after controlling normal distribution with Kolmogorov–Smirnov test. For normal distribution two-tailed paired student´s t-test was used and for not normal distribution Mann–Whitney test was used to calculate the significance. In case of multiple group comparisons, two-way ANOVA was used. Error bars indicate the standard deviation.

### Ethics approval and consent to participate

All experiments were performed in accordance with the German regulations and guidelines for animal experimentation. The study was approved by the “Regierung von Unterfranken” as the responsible authority (Permit Number 55.2-DMS-2532–2-42).

## Supplementary Information

Supplementary Information.

## Data Availability

All relevant data are in the paper and in the online supplement. Additional data, particularly on the DNA methylation pattern of the cell lines, will be provided upon request.

## Abbreviations

LSFM: Light-sheet-fluorescence-microscopy
FDA: Food and Drug Administration
VEGF: Vascular endothelial growth factor
PDGFR: Platelet derived growth factor
PI3K: Phosphoinositide-3-Kinase
STAT3: Signal transducer and activator of transcription 3
FCS: Fetal calf serum
