# Supplementary material for: Cytotoxic effects and tolerability of gemcitabine and axitinib in a xenograft model for c-myc amplified medulloblastoma
Source: Sci Rep. 2021 Jul 7;11:14062. doi: 10.1038/s41598-021-93586-x (PMC8263612; doi:10.1038/s41598-021-93586-x)
Supplement: Supplementary file 1 — Supplementary Information. [file 41598_2021_93586_MOESM1_ESM.pdf]

Supplemental Figure 1

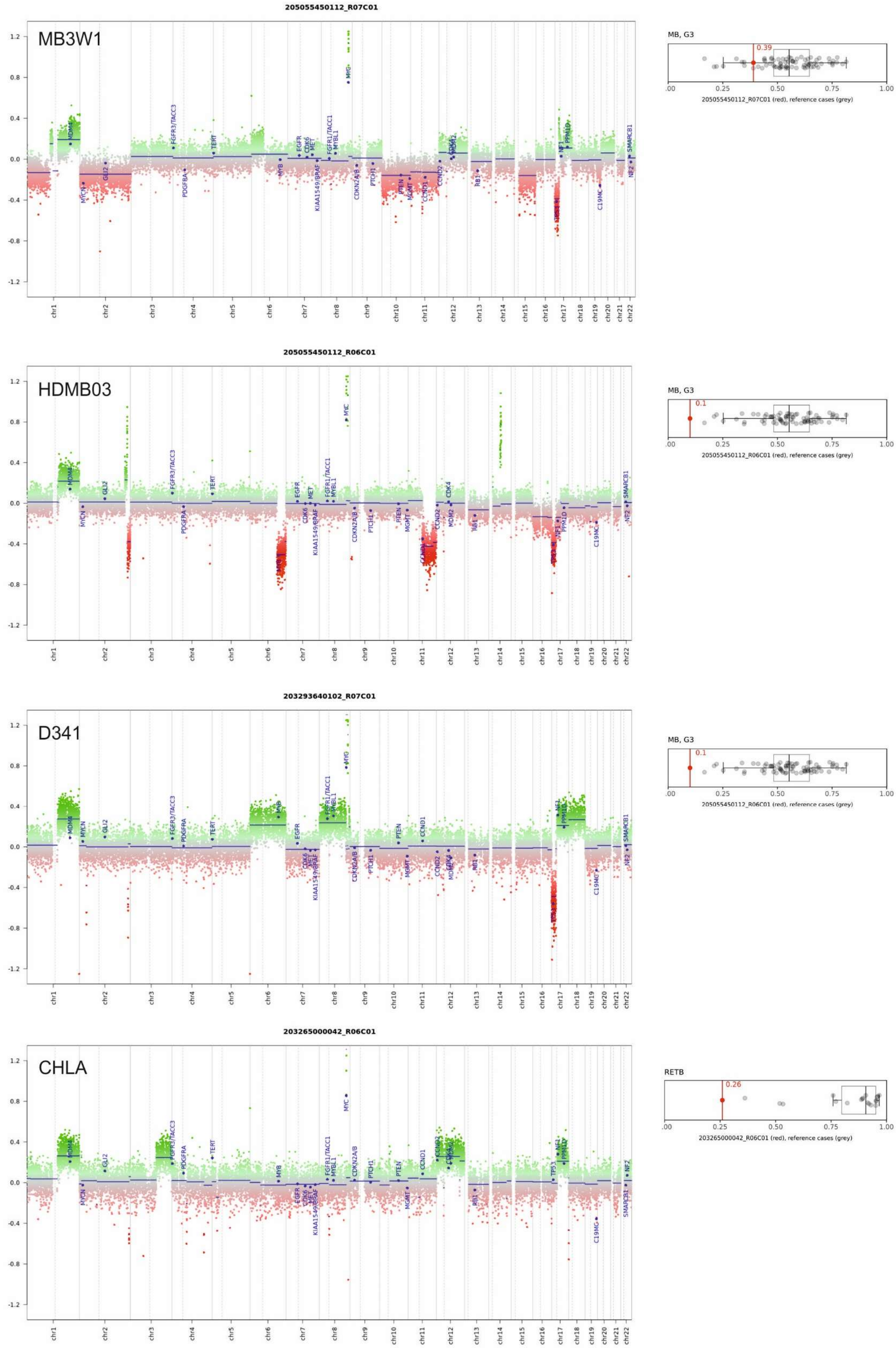

Supplemental Figure 1: The methylation data of the MB cell lines were categorized using the brain tumor classifier v11b4 (https://www.molecularneuropathology.org/mnp/classifier/2 (accessed on 6 April 2021) [Capper, D et al. DNA methylation-based classification of central nervous system tumours. Nature 2018, 555, 469–474.], which also generated a copy number variation (CNV) plot. MB3W1 cell line classified with an optimal calibrated score in the methylation class Medulloblastoma, Group 3 (MB, G3). HDMB03 and D341Med cell lines showed the highest raw classification scores in MB, G3 methylation class without reaching a calibrated score >0.3. CHLA cells tended to fall in the methylation class Retinoblastoma (RETB), in which they reached the maximum raw classification score and a calibrated score >0.3 (0.39) At CNV evaluation, all cell lines showed MYC amplification and 1q gain.

Supplemental Fig. 2

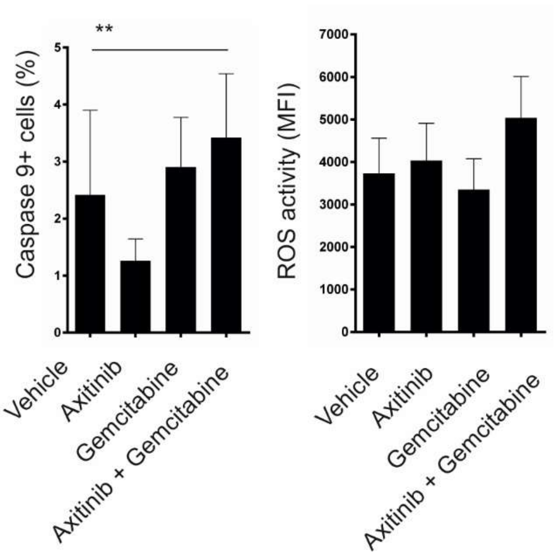

Supplemental Figure 2: Left: Caspase 9 analysis of MB3W1 after a 24h incubation with different cytostatics (EC50). Representative data from five independent experiments. (**p = .0089). Right: ROS activity in MB3W1 after 24h of incubation with cytostatics (EC50). Representative data are from five independent experiments.
